# Applicability of the Cox-Merz Rule to High-Density Polyethylene Materials with Various Molecular Masses

**DOI:** 10.3390/polym13081218

**Published:** 2021-04-09

**Authors:** Raffael Rathner, Wolfgang Roland, Hanny Albrecht, Franz Ruemer, Jürgen Miethlinger

**Affiliations:** 1 Institute of Polymer Extrusion and Compounding, Johannes Kepler University Linz, Altenberger Str. 69, 4040 Linz, Austria; wolfgang.roland@jku.at (W.R.); hanny.albrecht@Pro2Future.at (H.A.); juergen.miethlinger@gmail.com (J.M.); 2 Borealis Polyolefine GmbH, Sankt-Peter-Straße 25, 4021 Linz, Austria; franz.ruemer@borealisgroup.com

**Keywords:** Cox-Merz rule, high-viscosity HDPE materials, extrusion, modelling and simulation, rheology

## Abstract

The Cox-Merz rule is an empirical relationship that is commonly used in science and industry to determine shear viscosity on the basis of an oscillatory rheometry test. However, it does not apply to all polymer melts. Rheological data are of major importance in the design and dimensioning of polymer-processing equipment. In this work, we investigated whether the Cox-Merz rule is suitable for determining the shear-rate-dependent viscosity of several commercially available high-density polyethylene (HDPE) pipe grades with various molecular masses. We compared the results of parallel-plate oscillatory shear rheometry using the Cox-Merz empirical relation with those of high-pressure capillary and extrusion rheometry. To assess the validity of these techniques, we used the shear viscosities obtained by these methods to numerically simulate the pressure drop of a pipe head and compared the results to experimental measurements. We found that, for the HDPE grades tested, the viscosity data based on capillary pressure flow of the high molecular weight HDPE describes the pressure drop inside the pipe head significantly better than do data based on parallel-plate rheometry applying the Cox-Merz rule. For the lower molecular weight HDPE, both measurement techniques are in good accordance. Hence, we conclude that, while the Cox-Merz relationship is applicable to lower-molecular HDPE grades, it does not apply to certain HDPE grades with high molecular weight.

## 1. Introduction

The rheological behaviour of polymer melts is of major significance in polymer processing as it describes the deformation and flow behaviour of the material. A suitable choice of rheological model is essential for predicting the behaviour of a polymer during processing. Despite the wealth of publications in this context, the rheological behaviour of polymer melts remains a subject of scientific and technological interest [1,2,3] because it can be used to optimize a range of processing parameters and extrusion equipment. As it is influenced by a large number of parameters (e.g., concentration of the fluid, morphology, chemical structure), the rheological behaviour of polymer melts is very complex and sometimes difficult to relate to various physical properties of fluid polymer blends and alloys [4,5,6]. When determining the rheological behaviour of polymeric fluids, for which a variety of methods exist, the non-linear viscoelastic properties in particular give rise to high complexity. One approach—which is used, for example, in high-pressure capillary and extrusion rheometry—is to determine the rheological properties based on the pressure drop in a known geometry. Other methods are based on oscillatory measurements using, for instance, plate-plate and cone-plate rheometers. The big advantages of oscillatory over pressure-driven approaches are that they are fast, cheap, and easy to use [7]. Additionally, a relatively small amount of material is needed, and low shear rates can be measured. In comparison to purely rotational experiments, in oscillatory mode the imposed shear is relatively low, which avoids shear-induced heating of the test specimen [8]. It would thus be very useful to determine the rheological properties of polymers in oscillatory measurements. Cox and Merz [9] postulated an empirical relation, which states that the frequency dependence of the complex viscosity $\eta^{*}\left(\omega \right)$ of polystyrene melts with a range of molecular weights is equivalent to the shear rate dependence of the steady shear viscosity $\eta \left(\dot{\gamma }\right)$. Since then, the Cox-Merz rule has been investigated in the context of other polymers and was found to apply to various linear and branched polymers [10,11,12]. However, several research groups have reported that the Cox-Merz relation does not hold for some polymers (e.g., concentrated suspensions compounds, highly branched polymers, polymer blends, thermoplastic elastomers, functionalized polymers and in some cases high-molecular-weight polymers) [13,14,15,16,17,18]. For example, Snijkers and Vlassopoulos [19] found that the Cox-Merz relation did not apply to certain well-defined branched polymers they studied. Robertson and Roland [20] demonstrated the non-validity of the Cox-Merz rule for a variety of branched polyisobutylenes. Järvela [21] reported that the Cox-Merz rule does not apply to blends of polypropylene and maleated polypropylene. All three groups found several materials for which the Cox-Merz rule does not hold. Further reports [22,23] showed the non-validity of the Cox-Merz rule for filled polymers and materials that are able to form hydrogen bonds or exhibit other complex intermolecular binding phenomena (e.g., polyacrylamide and polyvinylchloride). Venkatraman and Okano [24] examined the applicability of the Cox-Merz rule to a variety of polyethylene types and found that it depends strongly on chemical structure, molecular weight, and entanglement. Additionally it has been shown that the Cox-Merz rule cannot be applied to certain low-density polyethylens (LDPE) and for random branched polystyrenes [25,26,27]. Another research group [28] stated that the Cox-Merz rule can be applied only in the low shear-rate region and that viscosity will be overestimated when the rule does not apply. Since the Cox-Merz rule cannot be applied in several cases, new postulations of the Cox-Merz rule have been made [29,30,31]. Overestimation or incorrect measurement of the viscosity has extreme consequences for the layout of processing tools, resulting, for instance, in incorrect pressure drops, residence time, and shear-rate distributions in the flow geometry, which can ultimately render the tools unusable.

Although there are no clear guidelines for when it can be applied, the Cox-Merz rule is widely used in the context of polyolefins in industrial practice because of the advantages of oscillatory measurements mentioned above. Since various studies have shown the non-validity of the Cox-Merz relation for particular kinds of polymers, we investigated whether it applies to commercially available—and in industry highly relevant—linear thermoplastic high-density polyethylene (HDPE) pipe grades. HDPE exhibits branching only to a very limited degree and consists of long chains that ensure its excellent mechanical properties [32]. To assess the suitability of the Cox-Merz rule for determining the viscosity of highly viscous HDPE pipe materials, we measured three commercially available polyethylene materials from Borealis using three well-known methods: (i) parallel-plate rheometry in oscillatory mode, (ii) high-pressure capillary rheometry, and (iii) extrusion rheometry. Based on the viscosity curves obtained, we then simulated the pressure drop along a pipe head and validated the results against experimental data.

## 2. Materials and Methods

### 2.1. Materials

In this study we used three commercially available HDPE grades:

- Material 1 was a high viscosity hexene copolymer polyethylene compound (HDPE) for pipe applications (PE 100) with high density and an outstanding resistance to slow crack growth.
- Material 2 was a high-density polyethylene for injection and compression moulding.
- Material 3 was another high-density polyethylene for injection and compression moulding.

Table 1 summarizes the melt flow rate (MFR) according to ISO 1133 (5.0 kg at 190 °C), mass average molecular weight Mw, and z-average molar mass Mz of the three different materials. Mw and Mz were measured with gel permeation chromatography (GPC).

### 2.2. Parallel-Plate Rheometer

The complex dynamic shear viscosity was measured by means of a combined motor-transducer (CMT) MCR302 rheometer from Anton Paar. The experiments were carried out at 200 °C under nitrogen atmosphere to prevent degradation, using a parallel-plate geometry with a diameter of 25 mm and a thickness of 0.8 mm. The frequency-sweep method with an amplitude of $\gamma_{0}$ = 0.03 was chosen to obtain frequency-dependent storage ($G^{\prime }$) and loss module ($G^{\prime \prime }$) for angular frequencies ω in the range between 0.01 rad/s and 628 rad/s. These modules were used to determine the shear-rate-dependent complex viscosity $\eta^{*}\left(\omega \right)$. The Cox-Merz relation was used to convert the oscillatory shear data into shear-rate-dependent viscosity $\eta \left(\dot{\gamma }\right)$:

(1) $$\eta \left(\dot{\gamma }\right)=\eta^{*}\left(\omega \right)$$

The complex viscosity $\eta^{*}$ was calculated by [33]:

(2) $$\left|\eta^{*}\right|=\frac{\left|G^{*}\right|}{\omega }\cdot \sqrt{1+{\frac{G^{\prime }}{G^{\prime \prime }}}^{2}}$$

where $G^{\prime \prime }$ is the loss modulus, $G^{\prime }$ the storage modulus, and ω the angular frequency. To determine the ratio of $G^{\prime }$ and $G^{\prime \prime }$, we calculated the phase shift (δ) by

(3) $$\delta =tan^{-1}\left(\frac{G^{\prime \prime }}{G^{\prime }}\right)$$

where a value of 45° indicates that *G′* and *G″* have the same value, and values above or below 45° indicate that the elastic part or the viscous part dominates, respectively.

### 2.3. High-Pressure Capillary Rheometer (HPCR)

A Rheograph 25 (Göttfert Werkstoff-Prüfmaschinen GmbH) high-pressure capillary rheometer with two different dies (diameter = 1 mm, length = 1 mm; diameter = 1 mm; length = 20 mm) was used to measure the polymer at a temperature of 200 °C. The shear viscosity was measured at shear rates between 1 and 5000 s^−1^. We calculated the wall shear stress $\tau_{w}$ according to [34]

(4) $$\tau_{w}=\frac{R}{2}\cdot -\frac{\Delta p}{l}$$

where R is the radius of the capillary, Δp the pressure drop, and l the length of the die capillary. The apparent shear rate ${\dot{\gamma }}_{app}$ was calculated by [34]

(5) $${\dot{\gamma }}_{app}=\frac{4\cdot \dot{V}}{\pi \cdot R^{3}}$$

where $\dot{V}$ is the volume flow through the capillary die. The Bagley correction [35] was used to correct for entry flow effects of the capillary. To obtain the true shear rate ${\dot{\gamma }}_{true}$ at the wall, we applied the Weissenberg–Rabinowitsch correction [36]:

(6) $${\dot{\gamma }}_{true}=\frac{1}{3}\cdot \left(2+\frac{dlog({\dot{\gamma }}_{app})}{dlog\left(\tau \right)}\right)\cdot {\dot{\gamma }}_{app}$$

The shear viscosity was then calculated by

(7) $$\eta =\frac{\tau_{w}}{{\dot{\gamma }}_{true}}$$

### 2.4. Slit-Die Extrusion Rheometer

A Thermo Haake Rheomex system consisting of a single-screw extruder (screw diameter 19 mm, length 33 times the diameter) equipped with a melt pump (2.4 cm^3^/rev.), a bypass valve, and a slit die with a defined gap height of 0.8 mm and a width of 20 mm was used to determine the shear-rate-dependent viscosity through the slit. The temperature profile was adjusted for the measurement to always reach a melt temperature of 200 °C at the entrance of the die for every measurement point.

We calculated the wall shear stress $\tau_{w}$ relative to the pressure drop Δp and the apparent shear rate ${\dot{\gamma }}_{app}$ relative to the volume flow $\dot{V}$ in a capillary slit of defined height H, length L, and width W according to [34]:

(8) $$\tau_{w}=\frac{\Delta p\cdot H}{2L}$$

(9) $${\dot{\gamma }}_{app}=\frac{\Delta p\cdot \dot{V}}{W\cdot H}$$

To obtain the true shear rate ${\dot{\gamma }}_{true}$ at the wall, we applied the Weissenberg–Rabinowitsch correction Equation (6) [36], and the shear viscosity was then calculated by Equation (7). The measurement range of the shear rate of the extrusion rheometer was between 25 and 300 s^−1^.

## 3. Simulation

### 3.1. Fitting of Experimental Data

The data obtained from oscillatory and capillary rheometry were fitted independently by using the ANSYS Polymat software module [37] for the modified Cross model [38] given by

(10) $$\eta \left(\dot{\gamma }\right)=\frac{\eta_{0}}{{\left(1+\lambda \cdot \dot{\gamma }\right)}^{m}}$$

where $\eta \left(\dot{\gamma }\right)$ is the shear viscosity, λ is the time constant [s], $\dot{\gamma }$ is the shear rate, $\eta_{0}$ is the zero shear rate viscosity, and m is the Cross-law flow behaviour index.

### 3.2. Simulation of Extrusion Equipment

**Figure 1:**
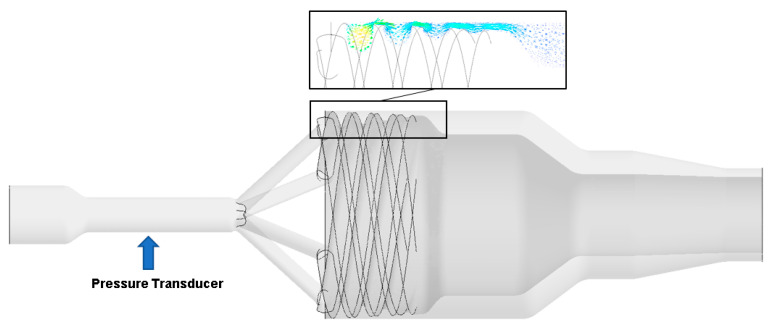
Real geometry of the experimentally validated and simulated pipe head.

Using the commercial finite-volume software ANSYS FLUENT [37], we simulated the HDPE melt flow in a pipe head. We assumed the flow to be (i) steady-state, (ii) creeping, (iii) incompressible, (iv) isothermal, and (v) we ignored gravity. We considered as the computational flow domain the full three-dimensional geometry of the spiral mandrel pipe head (Figure 1). A non-uniform mesh with 2,821,732 cells was generated for the numerical simulation and considered adequate for capturing the flow in the spiral distribution section.

At the inlet we specified the mass flow rate, and the pressure outlet was defined to have zero pressure. Further, we assumed that the fluid sticks to the die walls. We simulated five different setups with mass flowrates of 5, 10, 15, 20, and 25 kg/h.

All simulations were performed using a pressure-based coupled solver, and for the gradient computation we used the Green–Gauss node-based solver, which is recommended for an unstructured mesh. The second-order and second-order upwind schemes were employed to solve the pressure and momentum equations. Convergence was achieved when the scaled residual of mass conservation and momentum equations fell below 10^−5^. Subsequently, we evaluated the pressure drop of the flow geometry.

### 3.3. Extrusion Experiments with the Real Pipe Head

For the extrusion experiments we used a single screw extruder from ESDE with a diameter of 25 mm and a length of 18 D with a barrier screw and combined it with the pipe head shown in Figure 1. The pressure at the entrance of the pipe head was measured at the position indicated in Figure 1. The temperature profiles of the pipe head and extruder were adjusted to reach a melt temperature of 200 °C at the end of the pipe head. In accordance with the simulations the outputs were adjusted to 5, 10, 15, 20, and 25 kg/h for the experiments.

## 4. Results and Discussion

### 4.1. Comparison of Plate-Plate Rheometry (PPR) to High-Pressure Capillary Rheometry (HPCR) and Extrusion Slit Rheometry

**Figure 2:**
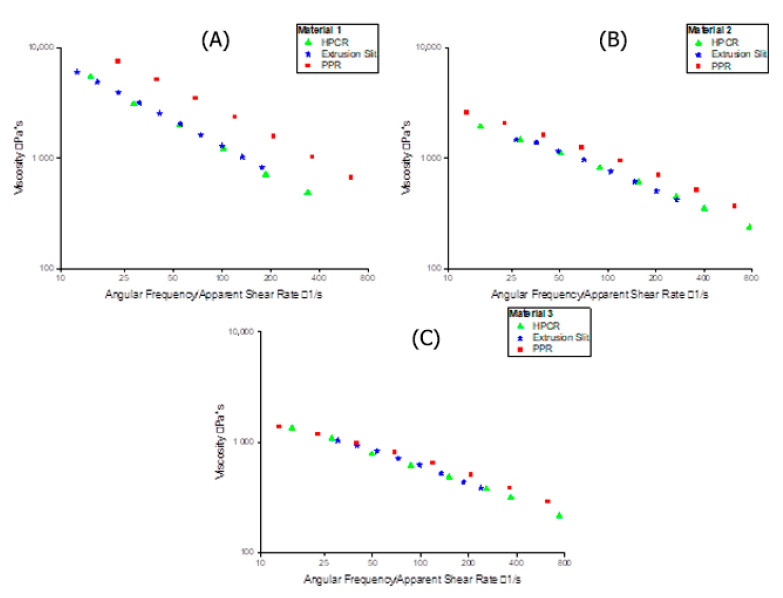
Comparison of pressure-flow-based measurements and oscillatory parallel-plate measurement of HDPEs at 200 °C. (**A**–**C**) show the data for Materials 1, 2, and 3, respectively.

**Figure 3:**
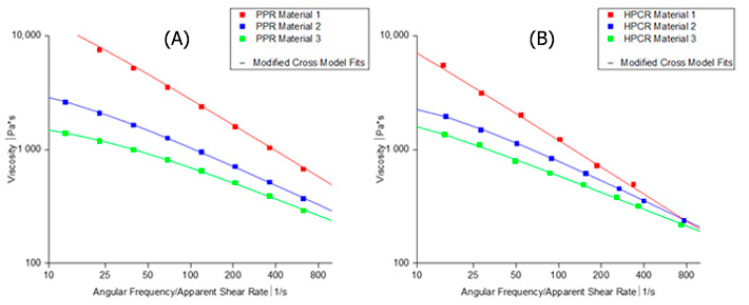
Comparison of the modified cross-law fits of Materials 1,2, and 3 with pressure-flow-based measurements (**B**) and oscillatory parallel-plate measurement (**A**) of HDPEs at 200 °C.

High-pressure capillary rheometry (HPCR) and extrusion slit rheometry measurements are both based on calculating the shear-rate-dependent viscosity via a pressure flow through a capillary die. Our comparison shows that the results of the two methods are in good accordance (see Figure 2). In contrast to HPCR and extrusion slit rheometry, which measure a constant pressure flow, plate-plate rheometry is based on oscillatory measurements. As can be seen in Figure 3, the two rheological approaches yield significantly different results for Materials 1 and 2, but are in good accordance for Material 3.

Since extrusion rheology and HPCR are almost identical, we carried out computational fluid dynamics (CFD) simulations only for HPCR and PPR data. The modified cross-law parameters for the three materials (derived from the PPR and HPCR measurements (Table 2 and Table 3)) were subsequently used to simulate the two methods. A comparison between the experimental data of PPR and HPCR with the modified cross model can be seen in Figure 3.

A comparison between the experimental viscosity data and the modified cross model is shown in Figure 3.

The cross-law flow behaviour index *m* is tends to unity for increasingly shear thinning behaviour. Indeed, only Material 3 indicates a Newtonian behaviour plateau with low *m* value. The zero shear viscosity ($\eta_{0})$ is strongly related to the *M_w_*. As $\eta_{0}$ increases so too does the molecular weight of the polymer. The modified cross-law parameters for an HDPE melt are strongly related to the mass average molecular weight *M_w_* according to Equations 11 and 13 [39]. For the tested materials, the exponents of the equations are listed in Table 4. The values are in good accordance to the literature [39].

(11) $$\eta_{0}\sim {\left[M_{w}\right]}^{\alpha }$$

(12) $$m\sim {\left[M_{w}\right]}^{\beta }$$

(13) $$\lambda \sim {\left[M_{w}\right]}^{\kappa }$$

To determine the difference in viscosities measured by PPR and HPCR at a range of shear rates, we calculated the viscosity ratios by:

(14) $$\varphi =\frac{\eta_{\mathrm{PPR}}}{\eta_{\mathrm{HPCR}}}$$

where $\eta_{\mathrm{PPR}}$ and $\eta_{\mathrm{HPCR}}$ are the viscosities measured at a particular shear rate by PPR and by HPCR, respectively.

From Table 5 it can be seen that the difference in rheological data measured by HPCR and PPR is immense for Material 1 and large for Material 2, whereas for Material 3 the two measurement techniques are in good accordance in the lower shear-rate region.

### 4.2. Viscoelasticity of HDPE Materials

**Figure 4:**
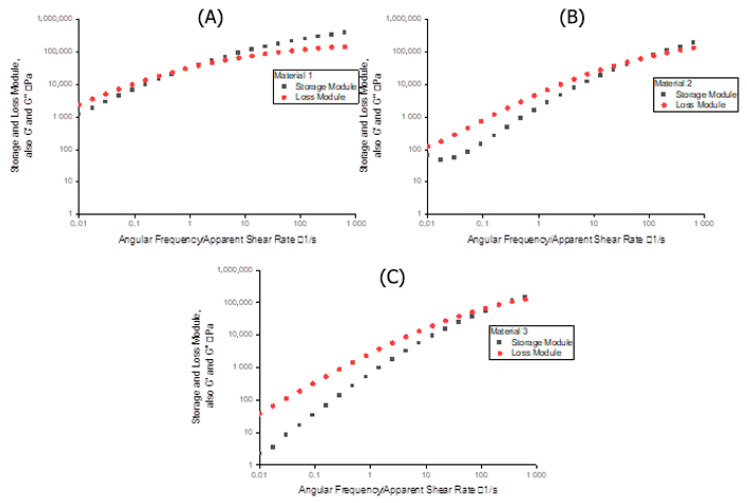
Storage and loss modules of three different HDPE materials. (**A**–**C**) show, respectively, the data of Materials 1, 2, and 3.

The storage and loss modules of the three different materials give insights into their elastic and viscous properties, respectively, and are illustrated in Figure 4.

For Material 1, the elastic part is more dominant between 10 s^−1^ and 400 s^−1^. For Material 2, the viscous part is more dominant up to 100 s^−1^, beyond which the elastic part becomes more dominant. For Material 3 the viscous part dominates between 10 s^−1^ and 400 s^−1^.

The cross-over point shifting to the low shear-rate region represents an increase in molecular mass. From Figure 4 it can be concluded that Material 1 has a higher average molecular weight than Material 2, which in turn has a higher average molecular weight than Material 3. Since the cross-over points of the three materials fall within a narrow range according to the storage and loss module, all three can be said to have similar molecular-weight distributions which is confirmed by the polydispersity index PI (Equation (15)). The PI for materials 1, 2 and 3 are 5.2, 5.0 and 4.5, respectively.

**Figure 5:**
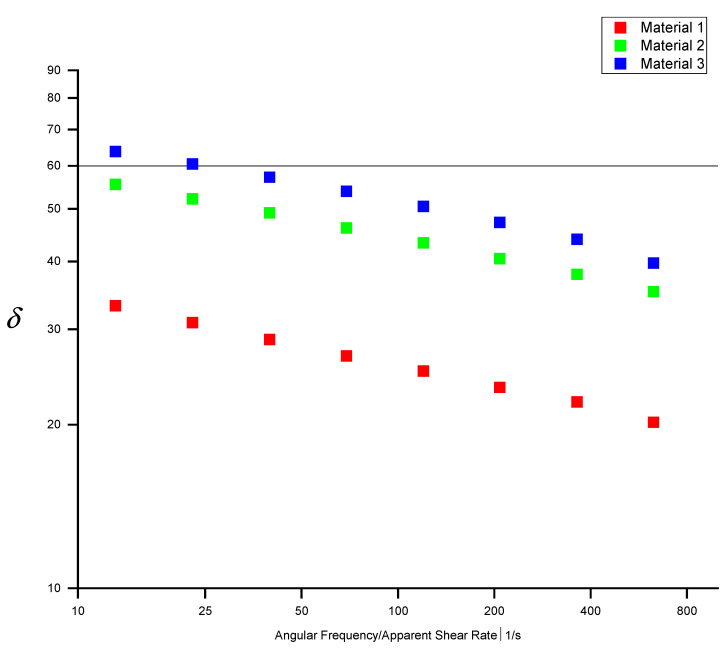
Phase shift of three different HDPE materials.

When the value of δ is close to 0° the material behaviour is elastic. If the value is close to 90° the material behaviour is viscous. From Figure 5 it can be seen that Material 1 exhibits the highest elastic behaviour and Material 3 the most viscous behaviour. The data in Figure 5 and Table 4 indicate that the Cox-Merz rule is applicable when the value of δ is 60° or higher. Below this value, the PPR and HPCR measurements start to differ significantly, and with decreasing phase shift the difference between PPR and HPCR increases.

(15) $$PI=\frac{M_{Z}}{M_{W}}$$

The cross-over points are in good correlation with the molecular masses from Table 1.

To investigate the influence of the elastic and viscous parts on the applicability of the Cox-Merz rule, we calculated the phase shift δ.

### 4.3. Comparison of Pipe-Head Simulations with Measured Rheology Curves

**Figure 6:**
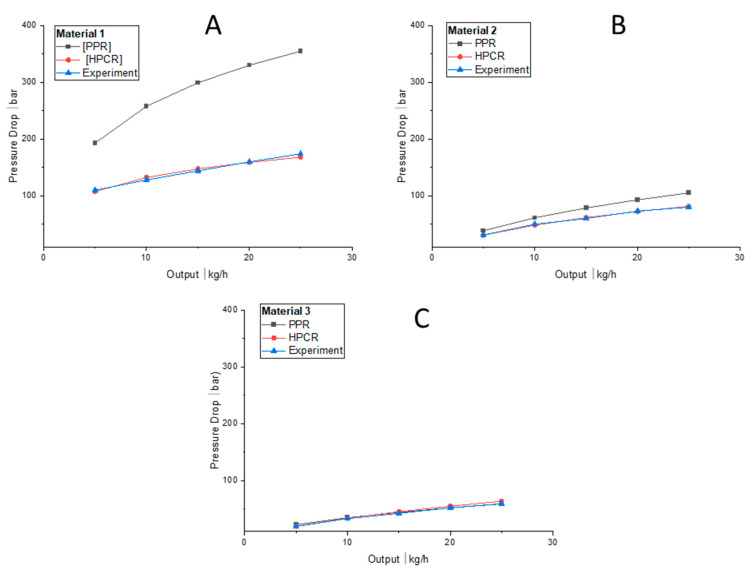
Comparison between the pressure drops according to two rheological models and experimental data at various mass flow rates for HDPE at 200 °C in a 32 mm pipe head. (**A**–**C**) show the simulation results for Materials 1, 2, and 3, respectively.

**Figure 7:**
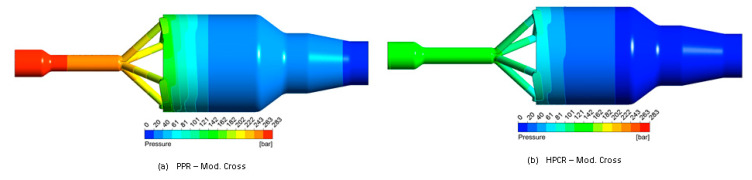
Pressure distribution in the pipe head for Material 1 at a mass flow rate of 10 kg/h.

Using the modified cross law to describe the shear-thinning flow behaviour with the parameters given in Table 2, we performed three-dimensional CFD simulations of the pipe head and evaluated the pressure drop along it—calculated as the difference between the area-weighted average pressures between the pressure transducer and the outlet—for the three different HDPE melts. Subsequently, we compared the results to experimental data measured at a range of flow rates (see Figure 6). For Material 1, the pressure drop in the pipe head is shown in Figure 7.

The ratio χ between the pressure drop simulated based on PPR data $p_{ppr}$ and the experimental data $p_{exp}$ was calculated by

(16) $$\chi =\frac{p_{ppr}}{p_{exp}}$$

The ratio ɕ between the pressure drop simulated based on HPCR data $p_{HPCR}$ and the experimental data was calculated by

(17) $$ɕ=\frac{p_{HPCR}}{P_{exp}}$$

The results are given in Table 6.

The results for Materials 1 and 2 show that the CFD simulations using PPR data overestimate the pressure drop, whereas the simulations based on HPCR data are in good accordance with the experiments. For Material 3, both the PPR-based and the HPTCR-based CFD simulations agree well with the experiments. As can be seen in Figure 6, the choice of measurement technique (PPR or HPCR) is indeed significant, and accurate experimental data is needed to yield good simulation results. Using incorrect rheological data for simulation may result in significant errors and consequently in equipment failure. For Material 1, the estimated pressure drop was twice as high as in the experiments, and for Material 2, the error was also substantial. The simulation results for Material 3, in contrast, show that there is no significant difference between the viscosities obtained.

## 5. Conclusions

We used three rheological measurement techniques to determine the shear-rate-dependent viscosity of three different HDPE materials: oscillatory parallel-plate, high-pressure capillary, and extrusion slit rheometry. While in parallel-plate rheometry the Cox-Merz relation is used to estimate the shear-dependent viscosity, in high-pressure capillary and extrusion slit rheometry the Weissenberg–Rabinowitsch relation is employed. Our data show that these methods differ significantly in accuracy depending on the material used. For Materials 1 and 2, PPR using the Cox-Merz relation overestimated the shear-rate-dependent viscosity significantly, whereas HPCR using the Bagley correction yielded results that accorded well with the experimental data (Figure 7). The applicability of the Cox-Merz rule to these materials depends heavily on the ratio between storage and loss module: If δ is 60° or greater, the Cox-Merz rule can be applied, while it becomes increasingly incorrect for decreasing values of δ. For Material 3, both HPCR- and PPR-based simulation results show good accordance with the rheological data from the experiments.

We conclude that the applicability of the Cox-Merz rule to HDPE materials is strongly dependent on the molecular mass of the material used. The polymers investigated differ significantly in molecular weight, and an increase in molecular weight resulted in considerable divergence from the Cox-Merz rule. The measured data of the very high-molecular-weight polymer Material 1 show a much greater difference between the shear viscosities obtained from PPR and HPCR than that of the data of the lower-molecular-weight Material 2, while for Material 3 no significant difference was observed. For HDPE—a long, straight polymer with limited branching and side chains—we thus conclude that with increasing molecular weight the disparity between the results from HPCR and PPR becomes significant. The Cox-Merz relation applies to HDPE only up to a particular molecular weight, more specifically up to an Mw of 85,000 according to our results.

Since viscosity data are essential to simulating the flow in various polymer-processing equipment, such as pipe heads and plasticizing screws, obtaining accurate viscosity curves is key to producing useful predictions. The consequence of using incorrect viscosity data in designing extrusion equipment is overestimation of the pressure drop in the die, which leads to completely different properties from those expected. Our results demonstrate that choosing the rheometry method according to the properties of the polymer of interest is crucial. In determining the viscosity of high-molecular-weight HDPE melts, capillary pressure flow is more reliable and accurate than is oscillatory measurement applying the Cox-Merz relation. The applicability of the Cox-Merz rule should also be examined critically for other long-chain polymers to ensure reliable rheological simulations when designing polymer-processing equipment.

## Figures and Tables

**Table 1:** Melt flow rate (MFR) and molecular weight distributions of the high-density polyethylene (HDPE) materials.

	MFR (g/10 min)	M_W_ (g/mol)	M_Z_ (g/mol)
Material 1	0.25	230,000	1,190,000
Material 2	1.5	110,000	550,000
Material 3	4.0	85,500	387,000

**Table 2:** Plate-plate rheometry (PPR)-based modified cross-law parameters for HDPE melt at 200 °C.

Parameter	Unit	Material 1	Material 2	Material 3
$\eta_{0}$	Pa.s	43,226	4559	1930
λ	s	0.362	0.125	0.0711
m	-	0.761	0.571	0.491

**Table 3:** High-pressure capillary rheometry (HPCR)-based modified cross-law parameters for HDPE melt at 200 °C.

Parameter	Unit	Material 1	Material 2	Material 3
$\eta_{0}$	Pa.s	56,796	3715	3175
λ	s	0.323	0.124	0.097
m	-	0.788	0.598	0.494

**Table 4:** Exponents of Equations (11)–(13).

	PPR	HPCR
*α*	3.09	3.12
*β*	0.43	0.45
*κ*	1.59	1.23

**Table 5:** Ratios of viscosities measured by PPR and HPCR at various shear rates $\dot{\gamma }$ for Materials 1–3.

	Material 1	Material 2	Material 3
$\dot{\gamma }$	φ	φ	φ
5	2.12	1.27	1.05
50	2.22	1.29	1.13
150	2.33	1.32	1.20
400	2.41	1.36	1.23

**Table 6:** Comparison of simulated pressure drops with experimental data at various output rates for Materials 1–3.

Output	Material 1		Material 2		Material 3	
kg/h	χ	ɕ	χ	ɕ	χ	ɕ
5	1.74	0.98	1.23	1	1.16	1
10	2.01	1.03	1.22	0.96	1.03	1
15	2.08	1.03	1.31	1.02	1.07	1.04
20	2.06	0.99	1.27	0.99	1	1.05
25	2.04	0.97	1.31	1.01	1	1.06

## Data Availability

The data presented in this study are available on request from the corresponding author.
